# Case report: Unveiling genetic and phenotypic variability in Nonketotic hyperglycinemia: an atypical early onset case associated with a novel *GLRX5* variant

**DOI:** 10.3389/fgene.2024.1432272

**Published:** 2024-09-11

**Authors:** Victor Marin, Louis Lebreton, Claire Guibet, Samir Mesli, Isabelle Redonnet-Vernhet, Mathurin Dexant, Delphine Lamireau, Sandrine Roche, Margaux Gaschignard, Jean Delmas, Henri Margot, Claire Bar

**Affiliations:** ^1^ Service de Biochimie, Groupe Hospitalier Pellegrin, CHU de Bordeaux, Bordeaux, France; ^2^ INSERM BRIC U1312 Université de Bordeaux, Bordeaux, France; ^3^ Hôpital Pédiatrique, Pôle Pédiatrique, CHU de Bordeaux, Bordeaux, France; ^4^ Service d’Imagerie Anténatale, de l’Enfant et de la Femme, Groupe Hospitalier Pellegrin, CHU de Bordeaux, Bordeaux, France; ^5^ Department of Medical Genetics, University of Bordeaux, MRGM INSERM U1211, CHU de Bordeaux, Bordeaux, France; ^6^ Service de Neurologie Pédiatrique, CHU Bordeaux, University Bordeaux, CNRS, INCIA, UMR 5287, NRGen Team, Bordeaux, France

**Keywords:** Nonketotic hyperglycinemia, NKH, GLRX5, glycine cleavage system, metabolic disorders, clinical genetics

## Abstract

Nonketotic hyperglycinemia (NKH) is a rare, autosomal recessive metabolic disorder usually associated with mutations in genes *AMT*, *GLDC* or *GCSH* involved in the glycine cleavage complex. Other genes have been linked with less severe NKH, associated with deficiency of lipoate cofactor such as *GLRX5, LIAS, BOLA3*. We identified a new case of GLRX5-mediated NKH who presented at 2-month with severe developmental delay and seizures. The initial suspicion was raised by the MRI and then confirmed by glycine measurements in cerebrospinal fluid and blood. Genetic analysis revealed a previously undescribed homozygous variant in the *GLRX5* gene [NM_016417.3:c.367G>C; p. (Asp123His)]. Despite medication and supportive care, he died at the age of 4 months after a sudden neurological deterioration. It was decided to limit therapeutic interventions due to the severity of the prognosis. The case was more severe than the previous GLRX5-mediated NKH described, regarding the early age at onset and the severity. Moreover, the genetic variant was located at a potentially crucial site for glutathione binding in the GLRX5 protein. This report, thereby, expands our understanding of NKH’s genetic underpinnings and phenotypic variability, highlighting the crucial role of *GLRX5* and other related genes in variant NKH.

## 1 Introduction

Nonketotic hyperglycinemia (NKH), also known as glycine encephalopathy is a rare inborn error of metabolism caused by an accumulation of the amino acid glycine in the body and particularly in the brain. Prevalence ranges from 1/63,000 in British Columbia, Canada to 1/9,684 in Kairouan where there is a highly consanguineous population (Nasrallah et al., 2020). The disease is attributed to a deficiency in the glycine cleavage system (GCS). Two forms of NKH are classically described in the literature: the severe form with neonatal onset, characterized by lethargy progressing to coma, apnea and seizures, which can lead to death, and the attenuated form with later onset, in which the developmental prognosis varies, with or without epilepsy (Krawiec and Anastasopoulou, 2023).

**FIGURE 1:**
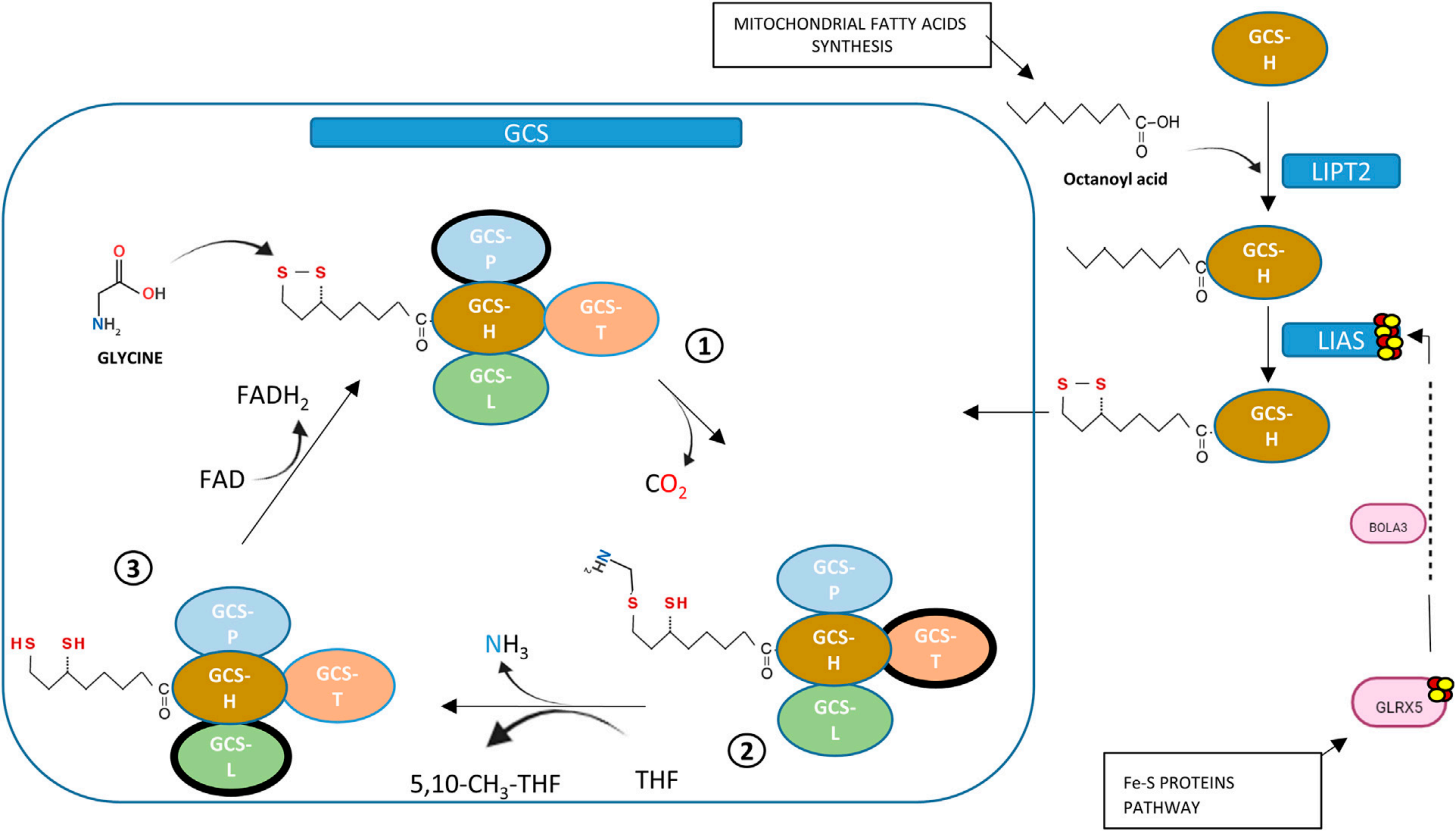
Links between GCS-lipoate synthesis and Fe-S proteins. Proteins from Fe-S pathway (GLRX5, BOLA3) are required for Iron-Sulphur Cluster synthesis (ISC). Mitochondrial fatty acid synthesis supplies octanoyl acid needed in lipoic acid formation. Lipoyl transferase (LIPT2) transfers octanoyl acid to H-protein of GCS in thioester form, then lipoyl synthase (LIAS), containing two [4Fe–4S], substitutes hydrogen for sulphur atoms, an activity that requires ISC supplied by GLRX5 and BOLA3. Lipoic acid bound to H-protein is essential for clivage of Glycine: glycine condensation with lipoate allows decarboxydation (step 1), transfer of methyl group to tetrahydrofolate to form 5,10-methylenetetrahydrofolateand release of ammonia (step 2). Last step of GCS is re-oxidation where dihydrolipoic acid is re-oxidized by dihydrolipoyl dehydrogenase using FAD+ (step 3).

Ninety-six percent of NKH cases are caused by mutations in three genes coding for components of the glycine cleavage system: *GLDC*, *AMT* and *GCSH*. There is a rarer entity recently described as “variant NKH” and linked to genes involved in the biosynthesis of iron-sulfur cluster (ISC) and lipoylation of GCS-H protein such as *GLRX5*, *BOLA3* and *LIAS* (Baker et al., 2014). The different steps of the biosynthesis of lipoic acid starting from octanoyl acid and involving enzymes like lipoyl synthase (LIAS) which incorporates sulfur atoms into the molecule are described in Figure 1 (Mayr et al., 2014; Cronan, 2020).

Here we describe the case of a newborn with a severe variant NKH caused by a previously unreported homozygous variant of the *GLRX5* gene and relate it to other cases documented in the scientific literature.

## 2 Materials and methods

### 2.1 Lactate dosage

Lactate levels were measured in both blood and cerebrospinal fluid (CSF) samples obtained from the patient. The measurements were conducted using the Abbott lactate assay kit (reference 9P18-21) following the manufacturer’s guidelines. The assay is based on the enzymatic conversion of lactate to pyruvate, coupled with the reduction of NAD + to NADH via spectrophotometry at 340 nm.

### 2.2 LC-MS/MS analysis of amino acids

Amino acids were analyzed in plasma, urine, and CSF samples and was performed on a ACQUITY Ultra Performance Liquid Chromtography system coupled with a Xevo TQ-S micro Tandem Quadrupole Mass Spectrometer (Waters, Manchester, United Kingdom). Prior to analysis, samples were derivatized using AccQ•Tag™reagents (Milford, MA, United States) according to the manufacturer’s instructions.

### 2.3 Urine organic acid profile

Urine organic acid detection and quantification were performed by gas chromatography–mass spectrometry Clarus 500™ (Perkin Elmer) after liquid/liquid extraction in ethyl acetate and derivatization by (N,O‐Bis [trimethylsilyl]trifluoroacetamide).

### 2.4 Next-generation sequencing (NGS)

For this study, genomic DNA was extracted from EDTA-treated whole blood samples. Two separate approaches were employed with a capture strategy: a targeted panel focusing on genes associated with inherited metabolic diseases, including hyperglycinemia-related genes such as *AMT, DLD, GLDC, GCSH*, and *GLRX5*, and whole exome sequencing. For both approaches, after purification and library preparation using Magnis Dx and Surselect kit XTHS2 (Agilent Technologies, Inc.) sequencing was performed on NextSeq 550 Dx (Illumina, San Diego, CA, United States), generating 2 × 75 bp paired-end reads. Base calling and primary analysis were performed via bcl2fastq (Illumina). Reads mapping, variant calling and annotation were conducted using the Alissa suite software (Agilent Technologies, Inc.). The pathogenicity of identified variants was assessed based on the criteria of the American College of medical genetic (ACMG) (Richards et al., 2015). To confirm the inheritance pattern of the identified variants, familial segregation studies were conducted via Sanger sequencing.

## 3 Case description

### 3.1 Clinical description

An 8-week-old male infant was referred to the pediatric neurology department due to concerns regarding hypotonia and abnormal movements. The infant’s mother reported early developmental issues, including a lack of smiles and visual contact, alongside clonic movements in the upper extremities since the first month of life. He was born full term, following a normal pregnancy. Notably, the parents were related through their grandparents, who were first cousins.

Clinical examination revealed significant axial hypotonia, poor visual tracking, and the presence of abnormal clonic movements in the upper extremities concomitant with upward deviation of the eyes. Video EEG showed disorganized background activity with multifocal spikes and waves and recorded episodes of spasms and focal seizures.

**FIGURE 2:**
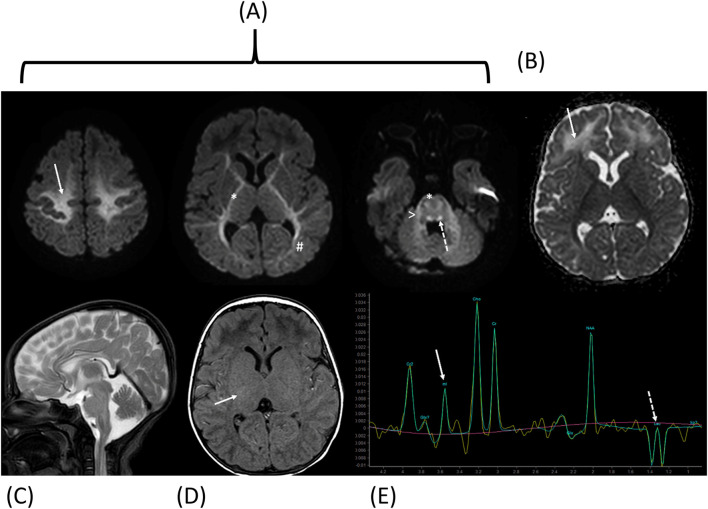
Cerebral magnetic resonance imaging and Proton spectroscopy at diagnosis. Diffusion-weighted imaging with Trace **(A)** and ADC map **(B)** shows restricted diffusion in the areas supposed to be myelinated [centrum semi-ovale (arrow), internal capsules and cortico-spinal tracts (*), optic radiations (#), dorsal brainstem (dotted arrow) and middle cerebellar peduncles (>)] consistent with intramyelinic edema, and vasogenic edemaof the frontal white matter (dotted arrow). Sagittal T2-weighted image **(C)** shows normal corpus callosum. Axial T1-weighted image **(D)** shows attenuated signal of the expected myelinated internal capsules (arrow). Proton spectroscopy **(E)** with intermediate echo time (144 ms) shows glycine peak (arrow; note that myo-inositol has a similar chemical shift but is not expected at 144 ms because of short T2 relaxation time) and lactate inverted doublet peaks (dotted arrow).

Cerebral magnetic resonance imaging demonstrated leukoencephalopathy with restricted diffusion in the areas presumed to be myelinated on diffusion-weighted imaging, including the centrum semi-ovale, internal capsules, corticospinal tracts, dorsal brainstem, and middle cerebellar peduncles, indicative of intramyelinic oedema, along with vasogenic oedema of the frontal white matter. Axial T1-weighted imaging showed attenuated signal of the expected myelinated internal capsules. Proton spectroscopy revealed glycine and lactate peaks, initially suggestive of NKH (Figure 2).

Serum and urine amino acid analysis revealed elevated levels of glycine, respectively 487 μmol/L (normal 160–240 μmol/L) and 2,693 μmol/L (normal 210–743 μmol/L). Lumbar puncture revealed elevated glycine level (45 μmol/L vs. normal 3–8.3 μmol/L) in the cerebrospinal fluid (CSF) with an increased glycine CSF/plasma ratio (0.092 vs. normal <0.02) (Krawiec and Anastasopoulou, 2023), suggestive of Nonketotic Hyperglycinemia. Interestingly, plasma lactate was slightly elevated (3.3 mmol/L vs. normal 0.5–2.2), with non-available plasma pyruvate level, and lumbar puncture revealed elevated lactate level (3.5 mmol/L vs. normal 1.2–1.9). Anti-epileptic treatment with levetiracetam, clobazam and a ketogenic diet reduced seizure frequency to 1 or 2 brief focal seizures per day. The strong suspicion of Nonketotic Hyperglycinemia prompted the introduction of dextromethorphan at 10 mg/kg/day in four divided doses alongside sodium benzoate at 250 mg/kg/day in four divided doses. Upon discharge from the hospital, the infant showed improved eye contact and tracking, along with enhanced head control.

The child presented to the emergency department twice within the following 2 weeks, due to a deterioration in general condition in the setting of febrile bloody diarrhea caused by *Campylobacter jejuni*. Both episodes resolved favorably with appropriate antibiotic therapy without modification of the underlying treatment regimen.

At 4 months of age, approximately 2 weeks after the resolution of the digestive symptoms, the child was admitted to the hospital because of a rapid onset of profound lethargy over a few hours with respiratory irregularities, without fever or any other symptom. Upon clinical examination, the child was stable hemodynamically, with a Glasgow Coma Scale score of 7 (E1,V1,M4), irregular breathing without desaturation. Blood tests revealed an elevated lactate level of 4.6 mmol/L, pH of 7.36, normocapnic, with no electrolyte disturbances or hepatic abnormalities. EEG findings showed markedly slowed background activity with abundant temporo-posterior sharp and wave discharges predominantly on the right, pseudo-rhythmic, without associated abnormal movements. Intravenous clonazepam therapy was attempted due to chewing movements, but there was no improvement in the level of consciousness. Despite low levels of glycine in both blood and urine, respectively 272 and 237 μmol/L, the dosages of dextromethorphan and sodium benzoate were increased, without clinical improvement. Respiratory viral PCR testing was negative. Given the severity of the condition, a decision was made to limit further aggressive therapies, and the patient died 4 days later from respiratory followed by cardiac arrest.

### 3.2 Genetic analysis

**FIGURE 3:**
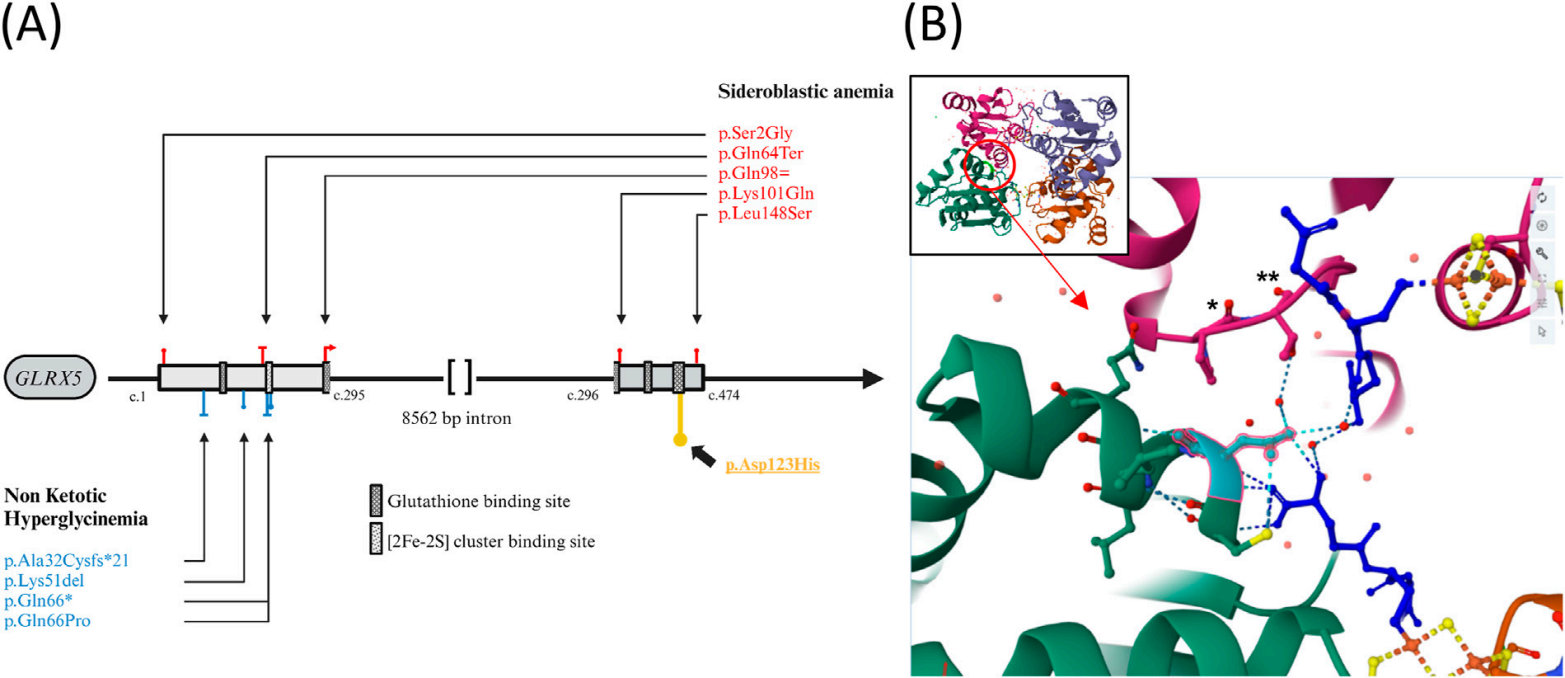
**(A)** Genetic Variations in the *GLRX5* Gene Associated with Sideroblastic Anemia (red) and Hyperglycinemia (blue). p.Asp123His described in this case is higlighted (yellow). Functional domains prediction from Uniprot.org **(B)** Visualization of the Interaction Site in GLRX5 Tetramer. Global view and focused view of the interaction site at position 123 (cyan) in a GLRX5 monomer (green). The second GLRX5 monomer is shown in pink. Glutathione are represented in deep blue. Fe-2S bridges are indicated in orange and yellow and oxygens are depicted in red. Variation Asp123His is predicted to result in the lost of several hydrogen bonds and provoke steric clash between His123 and Pro107(*) + Thr108 (**) of the interacting monomer [prediction via https://miztli.biokerden.eu/and Ittisoponpisan et al. (2019)]. The structure is visualized using the PDB file 2WUL on the rcsb.org website.

A comprehensive genetic analysis was conducted using a large gene panel for inborn metabolic genetic diseases, which included *AMT*, *DLD*, *GLDC*, *GCSH*, and *GLRX5* genes. This analysis led to the identification of a homozygous variation, c.367G>C; p. (Asp123His), located on exon 2 of the *GLRX5* gene (NM_016417.3) (Figure 3A). This variant was classified as “Likely pathogenic” according to the guidelines of the American Society for Medical Genetics and Genomics (ACMG) (Richards et al., 2015). Several factors support this classification, the identified variant is absent in the GnomAD (Karczewski et al., 2020) and ExAC (Karczewski et al., 2017) population databases (PM2 criteria). The nucleotide affected by this variant is highly conserved across different species, as evidenced by a phyloP score of 9.87 and *in silico* prediction algorithms unanimously predict its harmful effect, including CADD Phred (score: 31) (Rentzsch et al., 2019), REVEL (score: 0.675) (Ioannidis et al., 2016), AlphaMissense (0.998) (Cheng et al., 2023), and Mistic (score: 0.72) (Chennen et al., 2020) (PP3 criteria). The variant is situated within the critical Glutaredoxin protein domain (PM1 criteria) as depicted in Figure 3B, who shows that this protein is a tetramer constituted by 4 GLRX5 monomers. The variant Asp123 is located at the protein’s interfaces, interacting with glutathione and the neighboring side chain by hydrogen bonds. Furthermore, the patient exhibits a phenotype that is strongly correlated with a disease known to have a single genetic etiology (PP4 criteria).

We then validated the inheritance pattern of this variant by conducting familial segregation studies via Sanger sequencing for both parents which were found to harbor each a heterozygous copy of this variant. Whole exome sequencing analysis was performed to further explore potential genetic factors contributing to the unusually severe clinical presentation observed in the patient, retrieving no additional variant potentially involved but we cannot exclude the effect of unidentified intronic variants.

## 4 Discussion

We presented a case of non-ketotic hyperglycinemia diagnosed at 8 weeks of age in a child with a severe and early neurodevelopmental disorder associated with epilepsy, diagnosed by cerebral MRI and confirmed by metabolic screening and genetic analysis.

There is currently no curative treatment for NHK, and there is no consensus on the therapeutic strategy to be adopted. Sodium benzoate was used to reduce plasma glycine concentrations, with doses varying according to the glycine index (Van Hove et al., 2005). Dextromethorphan is another commonly used treatment because of its antagonistic effect on NMDA receptors, the hyperactivation of which has been implicated in glycine metabolism disorders (Van Hove et al., 2005). However, despite these interventions and reduced glycine levels, the patient’s condition suddenly deteriorated with no improvement despite increased dosages.

Several prognostic factors have been reported in the literature, such as age at first symptoms, CSF glycine concentration, CSF/plasma glycine ratio, and the presence of brain malformations on cMRI (Swanson et al., 2015, Kuseyri Hübschmann et al., 2022). In our case, the early onset of symptoms, severe developmental delay, and a CSF/blood glycine ratio >0.08 could have suggest a severe-like form of the disease but in newer classification (Kuseyri Hübschmann et al., 2022), our patient is in gray zone, as he does not align with most severe NKH cases (CSF/plasma ratio ≥0,15). But it is important to note that these classifications criteria are based on non-GLRX5 related NKH highlighting the need for tailored prognostic criteria.

Genetic testing revealed a previously undescribed homozygous pathogenic variant in *GLRX5.* This gene encodes a protein that plays a critical role in both iron-sulfur cluster synthesis and the lipoylation of the glycine cleavage system (Johansson et al., 2011). In March 2024, we identified in the Pubmed research a total of 8 probands with NKH related to the gene *GLRX5* (Baker et al., 2014; Chiong et al., 2007; Wei et al., 2011; Sankaran et al., 2021; Elliott et al., 2022; Anazi et al., 2017; Feng et al., 2021; Shamseldin et al., 2021)*.* Table 1 compiles the clinical and biological characteristics of these cases. The locations of the *GLRX5* variants associated with NKH are displayed in Figure 3A in comparison to the variants linked to congenital sideroblastic anemia (CSA) (Liu et al., 2014; Liu et al., 2016; Camaschella et al., 2007; Daher et al., 2019; Melo Arias et al., 2023).

**TABLE 1:** Clinical and biological characteristics of *GLRX5*-related NKH cases described.

Case	Our case	Feng et al. (2021)	Chiong et al. (2007)	Wei et al. (2011)	Baker et al. (2014)	Sankaran et al. (2021)	Sankaran et al. (2021)	Anazi et al. (2017)/Shamseldin et al. (2021)	Elliot et al. (2022)
DNA change 1	c.367G>C	c.196C>T	c.151_153del	c.151_153del	c.151_153del	c.151_153del	c.151_153del	c.197A>C	c.383T>C
Proteic change 1	p. (Asp123His)	p. (Gln66*)	p. (Lys51del)	p. (Lys51del)	p. (Lys51del)	p. (Lys51del)	p. (Lys51del)	p. (Gln66Pro)	p. (Met128Thr)
DNA change 2	c.367G>C	c.151_153del	c.151_153del	c.82insGCGTGCGG	c.151_153del	c.151_153del	c.151_153del	c.197A>C	c.383T>C
Proteic change 2	p. (Asp123His)	p. (Lys51del)	p. (Lys51del)	p. (Ala32Cysfs*21)	p. (Lys51del)	p. (Lys51del)	p. (Lys51del)	p. (Gln66Pro)	p. (Met128Thr)
Sex	M	F	F	M	F	F	F	M	F
Age of Onset	2m	1y3m	2y6m	1y6m	7y	3y	6y	1y	NA
Biologic features									
RBC abonormality	No	Hypochromic microcytic anemia	NA	NA	NA	NA	NA	NA	NA
Plasmatic Glycine (μmol/L)	204–487 (n 160–240)	812 (N: 20–760)	844 (N: 119–368)	829	804 (N:119–368)	586–705 (N:119–368)	730–1,224 (N:119–368)	NA	NA
CSF Glycine (µmol/L)	45	NA	23 (N: 3–9)	24.8	15 (N: 3–9)	28 (N: 3–9)	53 (N: 3–9)	NA	NA
CSF/Plasma ratio (≤0.02)	0.092	NA	0.027	0.03	0.02	0.04	0.04	NA	NA
Lactate (<2 mmol/L)	2.9	3.47	0.2–1.8	1.2	0.6–2.6	NA	NA	NA	NA
CSF Lactate	3.5	NA	1.4–1.5	1.25	1.4–1.5	NA	NA	NA	NA
Clinical features									
Neurologic	Hypotonia, clonic movements, seizures	Quick deterioration of language and mouvement abilities, spastic paralysis, reduced muscle strength	Gait disturbance, spastic paraparesis	Gait disturbance, nystagmus, spastic paraparesis	Increasing spastic paraparesis and clumsiness, no cognitive impairement	Gait difficulty, wheelchair-bound at 30, obsessive-compulsive disorder	Falls, seizures, low intellectual function	Hypotonia, seizures, developmental delay, microcephaly, severe hypotonia, spacticity	NA
cMRI	Diffuse intramyelinic edema with reactional vasogenic edema	Multifocal T2 increased signal in brain and spine, cystic white matter lesions	Leukodystrophy	Leukodystrophy and extensive medulla lesion	Spinal cord lesions	Normal (49y)	Normal (38y)	Diffuse hyperintensity, thalamic atrophy, bilateral periventricular necrosis	NA
Opthalmologic	Abnormal eye tracking	Leopard skin-like fundus oculi and bilateral optic atrophy	NA	Optic atrophy and strabismus	Mild pallor of the discs	Bilateral optic disc pallor	Severe disc pallor-atrophy	Blindness	NA
Treatment	Sodium Benzoate Dextrometorphan	Baclofen, nitrazepam, trihexyphenidyl, B1, B2, C, E, CoQ10, B8, Iron dextran, Lipoic acid and 5-ALA Improved outcome	Sodium benzoate 250 mg/kg/day Protein restriction <1.5 g/kg/day Botulinum toxin injections	No treatment	Botulinum toxin injections α-lipoic acid (300 mg twice daily) no improvment	Protein restricted diet Sodium Benzoate	NA	NA	NA
Outcome	Died 4m	Died 4y (infection)	Alive 20y	Alive 7y (neurological defect)	Alive 19y	Alive 56y	Alive 45y	Died 30m	NA

Notably, our case presented with the earliest onset *GLRX5* related NKH and the shortest lifespan (only 4 months), compared to the second most severe case reported (Anazi et al., 2017; Shamseldin et al., 2021), who survived until the age of 30 months. Our patient had urine and blood glycine concentrations that were slightly elevated but relatively lower than the ones given in the literature. However, our case presented the highest reported CSF/plasma glycine ratio to date, underlying the importance of this ratio in assessing disease diagnosis and severity. This case is among the very few documented cases of early-life mortality associated with *GLRX5*-related NKH.

The genetic variant identified in our case is located in the interaction site with glutathione. The histidine substitution is predicted to disrupt the hydrogen bonds with glutathione and alter the 3D configuration of the GLRX5 tetramer by interfering with the interactions between GLRX5 monomers (Figures 3A, B) (Ittisoponpisan et al., 2019). Another variant at the same amino acid position has been identified in GnomAD and dbSNP (rs748991738), although no homozygous carriers of this variant are known and impact on 3D structure seems to be less important. Glutathione is considered crucial for GLRX5’s function, particularly in its role in binding to iron-sulfur complexes (Olive and Cowan, 2018).The other severe case described (Anazi et al., 2017; Shamseldin et al., 2021) involved a missense variant replacing glutamine with proline at position 66, which could disrupt the binding region of these iron-sulfur complexes [2FE-2S] located at amino acid position 67. These two cases contrasts with others carrying the recurrent p. (Lys51del) variant, which has been identified in at least 6 patients with a notably higher life expectancy, potentially exerting a milder impact.

Lipoic acid is also a cofactor of pyruvate dehydrogenase (PDH), and its deficiency due to GLRX5 disruption could theoretically lead to hyperlactatemia (Mayr et al., 2014). However, lactate elevation was inconsistent among reported patients. It has been demonstrated that the specific nature of GLRX5 variants can influence PDH activity (Liu et al., 2016), and even patients with the same genotype can exhibit different lactate levels (Table 1). Moreover, hyperlactatemia can result from other factors such as difficulties in sample collection in children, seizures, and more. Further studies and cases reported will help to elucidate if the level of lactate reflect the PDH dysfunction and can be a prognostic marker for the GLRX5 related NKH.

In addition to NKH (OMIM 616860), pathogenic variants in the GLRX5 gene have also been associated with CSA (OMIM 616859) (Sankaran et al., 2021; Ye et al., 2010). We have not found any variant involved in both phenotypes, nor any disease specific hotspot, and the impact of the variant on the protein (loss of function or missense) does not seem to determine the occurrence of either pathology (Figure 3A). It is noteworthy that the pathophysiological causes of Nonketotic Hyperglycinemia (NKH) and Congenital Sideroblastic Anemia (CSA) differ. NKH results from a defect in the final step of lipoic acid synthesis, which involves the transfer of two sulfur atoms from the 4Fe-4S complex to lipoic acid. On the other hand, CSA is caused by a reduction in the intracellular concentration of the 4Fe-4S cluster, leading to an increased concentration of non-complexed Iron Regulatory Protein 1 (IRP1) (Liu et al., 2016). IRP1 is a cytosolic aconitase that, when not bound to an iron-sulfur (Fe-S) cluster, functions as an iron regulatory protein. Non-complexed IRP1 binds to iron-responsive elements (IREs) in the mRNA of several genes, including 5-Aminolevulinic Acid Synthase 2 (ALAS2), the rate-limiting enzyme in heme synthesis, leading to its repression. This increase ultimately results in the repression of ALAS2 expression and disrupts the heme synthesis pathway. The distinct genetic variations observed in these conditions suggest separate genetic mechanisms. Our patient did not show signs of CSA on blood exploration, although bone marrow analysis was not conducted, leaving this aspect inconclusive. Future genetic analyses and experimental research are essential to further elucidate these mechanisms.

## 5 Conclusion

In this case report, we present an exceptionally severe and atypical case of NKH associated with a novel homozygous variant in *GLRX5* gene [c.367G>C; p. (Asp123His)]. The patient’s condition rapidly deteriorated, leading to a tragic outcome at a very young age.

Despite aggressive medical management, including glycine-lowering and anti-epileptic therapies, the patient’s clinical status worsened precipitously. This rapid deterioration underscored the challenges in managing such severe cases of NKH.

The identification of a previously unreported homozygous variant in the *GLRX5* gene raises intriguing questions about its potential impact on protein function and its role in the severity of the disease phenotype. While further research is needed to elucidate the precise mechanisms involved, this case highlights the significance of considering rare and unique variants in understanding the genetic basis of severe NKH cases. Such discoveries may contribute to improved diagnostics and therapies for individuals with this rare disorder.

## Data Availability

The original contributions presented in the study are included in the article/supplementary material, further inquiries can be directed to the corresponding author.

## References

[B1] Anazi S. Maddirevula S. Salpietro V. Asi Y. T. Alsahli S. Alhashem A. (2017). Expanding the genetic heterogeneity of intellectual disability. Hum. Genet. 136 (11-12), 1419–1429. 10.1007/s00439-017-1843-2 28940097

[B2] Baker P. R. Friederich M. W. Swanson M. A. Shaikh T. Bhattacharya K. Scharer G. H. (2014). Variant non ketotic hyperglycinemia is caused by mutations in LIAS, BOLA3 and the novel gene GLRX5. Brain. 137 (Pt 2), 366–379. 10.1093/brain/awt328 24334290 PMC3914472

[B3] Camaschella C. Campanella A. De Falco L. Boschetto L. Merlini R. Silvestri L. (2007). The human counterpart of zebrafish shiraz shows sideroblastic-like microcytic anemia and iron overload. Blood. 110 (4), 1353–1358. 10.1182/blood-2007-02-072520 17485548

[B4] Cheng J. Novati G. Pan J. Bycroft C. Žemgulytė A. Applebaum T. (2023). Accurate proteome-wide missense variant effect prediction with AlphaMissense. Science 381, eadg7492. 10.1126/science.adg7492 37733863

[B5] Chennen K. Weber T. Lornage X. Kress A. Böhm J. Thompson J. (2020). MISTIC: a prediction tool to reveal disease-relevant deleterious missense variants. PLoS One. 15 (7), e0236962. 10.1371/journal.pone.0236962 32735577 PMC7394404

[B6] Chiong M. A. Procopis P. Carpenter K. Wilcken B. (2007). Late-onset nonketotic hyperglycinemia with leukodystrophy and an unusual clinical course. Pediatr. Neurol. 37 (4), 283–286. 10.1016/j.pediatrneurol.2007.05.016 17903674

[B7] Cronan J. E. (2020). Progress in the enzymology of the mitochondrial diseases of lipoic acid requiring enzymes. Front. Genet. 11, 510. 10.3389/fgene.2020.00510 32508887 PMC7253636

[B8] Daher R. Mansouri A. Martelli A. Bayart S. Manceau H. Callebaut I. (2019). GLRX5 mutations impair heme biosynthetic enzymes ALA synthase 2 and ferrochelatase in Human congenital sideroblastic anemia. Mol. Genet. Metab. 128 (3), 342–351. 10.1016/j.ymgme.2018.12.012 30660387

[B9] Elliott A. M. Adam S. du Souich C. Lehman A. Nelson T. N. van Karnebeek C. (2022). Genome-wide sequencing and the clinical diagnosis of genetic disease: the CAUSES study. HGG Adv. 3 (3), 100108. 10.1016/j.xhgg.2022.100108 35599849 PMC9117924

[B10] Feng W. X. Zhuo X. W. Liu Z. M. Li J. W. Zhang W. H. Wu Y. (2021). Case report: a variant non-ketotic hyperglycinemia with GLRX5 mutations: manifestation of deficiency of activities of the respiratory chain enzymes. Front. Genet. 12, 605778. 10.3389/fgene.2021.605778 34054912 PMC8155699

[B11] Ioannidis N. M. Rothstein J. H. Pejaver V. Middha S. McDonnell S. K. Baheti S. (2016). REVEL: an ensemble method for predicting the pathogenicity of rare missense variants. Am. J. Hum. Genet. 99, 877–885. 10.1016/j.ajhg.2016.08.016 27666373 PMC5065685

[B12] Ittisoponpisan S. Islam S. A. Khanna T. Alhuzimi E. David A. Sternberg M. J. E. (2019). Can predicted protein 3D structures provide reliable insights into whether missense variants are disease associated? J. Mol. Biol. 431 (11), 2197–2212. 10.1016/j.jmb.2019.04.009 30995449 PMC6544567

[B13] Johansson C. Roos A. K. Montano S. J. Sengupta R. Filippakopoulos P. Guo K. (2011). The crystal structure of human GLRX5: iron-sulfur cluster co-ordination, tetrameric assembly and monomer activity. Biochem. J. 433 (2), 303–311. 10.1042/BJ20101286 21029046

[B14] Karczewski K. J. Francioli L. C. Tiao G. Cummings B. B. Alföldi J. Wang Q. (2020). The mutational constraint spectrum quantified from variation in 141,456 humans. Nature 581 (7809), 434–443. 10.1038/s41586-020-2308-7 32461654 PMC7334197

[B15] Karczewski K. J. Weisburd B. Thomas B. Solomonson M. Ruderfer D. M. Kavanagh D. (2017). The ExAC browser: displaying reference data information from over 60 000 exomes. Nucleic Acids Res. 45 (Database issue), D840–D845. 10.1093/nar/gkw971 27899611 PMC5210650

[B16] Krawiec C. Anastasopoulou C. (2023). “Nonketotic hyperglycinemia,” in StatPearls (Treasure Island (FL): StatPearls Publishing). 32310600

[B17] Kuseyri Hübschmann O. Juliá-Palacios N. A. Olivella M. Guder P. Zafeiriou D. I. Horvath G. (2022). Integrative approach to predict severity in nonketotic hyperglycinemia. Ann. Neurol. 92 (2), 292–303. 10.1002/ana.26423 35616651

[B18] Liu G. Guo S. Anderson G. J. Camaschella C. Han B. Nie G. (2014). Heterozygous missense mutations in the GLRX5 gene cause sideroblastic anemia in a Chinese patient. Blood 124 (17), 2750–2751. 10.1182/blood-2014-08-598508 25342667

[B19] Liu G. Wang Y. Anderson G. J. Camaschella C. Chang Y. Nie G. (2016). Functional analysis of GLRX5 mutants reveals distinct functionalities of GLRX5 protein. J. Cell Biochem. 117 (1), 207–217. 10.1002/jcb.25267 26100117

[B20] Mayr J. A. Feichtinger R. G. Tort F. Ribes A. Sperl W. (2014). Lipoic acid biosynthesis defects. J. Inherit. Metab. Dis. 37 (4), 553–563. 10.1007/s10545-014-9705-8 24777537

[B21] Melo Arias A. F. Escribano Serrat S. Martínez Nieto J. Medina Salazar F. Ropero Gradilla P. Benavente Cuesta C. (2023). Two new mutations in the GLRX5 gene cause sideroblastic anemia. Blood Cells, Mol. Dis. 102, 102763. 10.1016/j.bcmd.2023.102763 37301020

[B22] Nasrallah F. Hadj-Taieb S. Chehida A. B. Jelassi A. Ben Massoued S. Charfi M. (2020). Nonketotic hyperglycinemia in Tunisia. Report upon a series of 69 patients. Neuropediatrics 51 (5), 349–353. 10.1055/s-0040-1712489 32818969

[B23] Olive J. A. Cowan J. A. (2018). Role of the HSPA9/HSC20 chaperone pair in promoting directional human iron-sulfur cluster exchange involving monothiol glutaredoxin 5. J. Inorg. Biochem. 184, 100–107. 10.1016/j.jinorgbio.2018.04.007 29689452 PMC5964037

[B24] Rentzsch P. Witten D. Cooper G. M. Shendure J. Kircher M. (2019). CADD: predicting the deleteriousness of variants throughout the human genome. Nucleic Acids Res. 47 (D1), D886–D894. 10.1093/nar/gky1016 30371827 PMC6323892

[B25] Richards S. Aziz N. Bale S. Bick D. Das S. Gastier-Foster J. (2015). Standards and guidelines for the interpretation of sequence variants: a joint consensus recommendation of the American College of medical genetics and genomics and the association for molecular pathology. Genet. Med. 17 (5), 405–424. 10.1038/gim.2015.30 25741868 PMC4544753

[B26] Sankaran B. P. Gupta S. Tchan M. Devanapalli B. Rahman Y. Procopis P. (2021). GLRX5-associated [Fe-S] cluster biogenesis disorder: further characterisation of the neurological phenotype and long-term outcome. Orphanet J. Rare Dis. 16 (1), 465. 10.1186/s13023-021-02073-z 34732213 PMC8565018

[B27] Shamseldin H. E. AlAbdi L. Maddirevula S. Alsaif H. S. Alzahrani F. Ewida N. (2021). Lethal variants in humans: lessons learned from a large molecular autopsy cohort. Genome Med. 13 (1), 161. 10.1186/s13073-021-00973-0 34645488 PMC8511862

[B28] Swanson M. A. Coughlin C. R. Scharer G. H. Szerlong H. J. Bjoraker K. J. Spector E. B. (2015). Biochemical and molecular predictors for prognosis in nonketotic hyperglycinemia. Ann. Neurol. 78 (4), 606–618. 10.1002/ana.24485 26179960 PMC4767401

[B29] Van Hove J. L. K. Vande Kerckhove K. Hennermann J. B. Mahieu V. Declercq P. Mertens S. (2005). Benzoate treatment and the glycine index in nonketotic hyperglycinaemia. J. Inherit. Metab. Dis. 28 (5), 651–663. 10.1007/s10545-005-0033-x 16151895

[B30] Wei S. H. Weng W. C. Lee N. C. Hwu W. L. Lee W. T. (2011). Unusual spinal cord lesions in late-onset non-ketotic hyperglycinemia. J. Child. Neurol. 26 (7), 900–903. 10.1177/0883073810393965 21471552

[B31] Ye H. Jeong S. Y. Ghosh M. C. Kovtunovych G. Silvestri L. Ortillo D. (2010). Glutaredoxin 5 deficiency causes sideroblastic anemia by specifically impairing heme biosynthesis and depleting cytosolic iron in human erythroblasts. J. Clin. Invest. 120 (5), 1749–1761. 10.1172/JCI40372 20364084 PMC2860907

